# All talk, little action: A scoping review mapping lifelong learning education strategies in postgraduate medical education

**DOI:** 10.4102/jcmsa.v4i1.427

**Published:** 2026-07-23

**Authors:** Megan O’Connor, Antoine G.L. Rocher, Jennifer M. Wolf, Veena Singaram, Leonard C. Marais

**Affiliations:** 1 Discipline of Orthopaedic Surgery, School of Clinical Medicine, University of KwaZulu-Natal, Durban, South Africa; 2 Department of Orthopaedic Surgery and Rehabilitation, Faculty of Medicine, University of Chicago, Chicago, Illinois, United States of America; 3 Academic Leader of Research, School of Clinical Medicine, University of KwaZulu-Natal, Durban, South Africa

**Keywords:** lifelong learning, postgraduate medical education, registrars, scoping review, education strategies, goal-setting, reflective practice, evidence-based medicine

## Abstract

**Background:**

Lifelong learning (LLL) is a core competency in postgraduate medical education (PGME), yet little is known about how registrar programmes teach it. Although LLL is prominent in policy and discourse, knowledge of best-practice educational strategies during registrar training remains limited.

**Aim:**

This study aimed to map and synthesise the empirical research of strategies designed to foster LLL in registrar training.

**Setting:**

The study was carried out at postgraduate orthopaedic training institutions in South Africa.

**Method:**

A scoping review was conducted in accordance with the preferred reporting items for systematic reviews and meta-analyses extension for scoping review (PRISMA-ScR) guidelines to identify educational strategies that promote LLL in PGME. Searches of MEDLINE, EMBASE, Web of Science, and the Cochrane Library were performed from database inception to November 2025 using medical subject headings (MeSH) terms including ‘lifelong learning’ and ‘registrar’, supplemented by reference screening. Bibliographic data were tabulated, and strategies synthesised into three overarching categories.

**Results:**

Of the 492 records screened, eight studies met the inclusion criteria. Strategies clustered into three groups: (1) goal-setting; (2) reflection, self-assessment, and feedback; and (3) evidence-based medicine (EBM) training. The vehicles for delivering these strategies included individualised learning plans, portfolios, goal-setting requirements, and EBM training courses.

**Conclusion:**

The review identified eight studies describing strategies to foster LLL in registrar training, concentrated in North America, and non-surgical disciplines. The paucity and heterogeneity of published evidence highlight the need for intentional incorporation and evaluation of the impact of LLL in postgraduate curricula across diverse contexts.

**Contribution:**

This review provides the first mapped synthesis of explicit LLL educational strategies in PGME, identifying gaps and informing future curriculum design.

## Introduction

Lifelong learning (LLL) is widely recognised as a cornerstone of medical professionalism, enabling clinicians to adapt to rapidly evolving medical knowledge and practice.^1,2^ Beyond sustaining clinical competence, LLL is considered a marker of curriculum quality in postgraduate medical education (PGME)^3^ and a core value in speciality training.^4^ Its importance is underscored by the reality that in the fourth industrial revolution, powerful artificial intelligence (AI) systems still struggle with tasks that require adaptive reasoning and reflective growth, skills essential for safe and effective medical practice.^5^

Despite its prominence in policy and discourse, how LLL is intentionally taught during PGME is less well defined.^6^ While strategies such as problem-based learning, self-directed learning (SDL), and evidence-based medicine (EBM) are well documented to promote LLL in undergraduate literature,^7,8^ and continuing medical education (CME), continuing professional development (CPD),^9,10^ and maintenance of certification (MOC) programmes operationalise LLL in specialist practice,^1,11^ the specialist training period appears to receive less emphasis with regard to deliberate instruction in LLL.^6^ Possible reasons include the belief that undergraduate-acquired LLL skills endure,^8^ the perception that residency curricula leave little room beyond clinical and procedural requirements,^6^ and the assumption that LLL habits are developed naturally during training rather than through deliberate teaching.^12^

Existing research in PGME has focused mainly on defining and measuring LLL, rather than on developing educational strategies. Hojat et al. defined LLL as ‘self-initiated activities and information-seeking skills activated in individuals with sustained motivation to learn and the ability to recognise their own learning needs’.^13^ Conway reasons that definition is a critical step to ensure clarity in curriculum design, recognising the need for distinction between often conflated concepts such as self-regulated learning.^14^ Building on Hojat et al.’s definition, validated assessment tools such as the Jefferson Scale of Physician Lifelong Learning have been developed and compared for efficacy.^15,16^ These measures have been applied across the medical education continuum, revealing that registrars in training represent the most variable group in terms of LLL orientation.^17^ In fact, Ortwein et al. found that registrars place less value on LLL compared to specialists,^18^ which may contribute to registrars’ lack of readiness for independent practice.^11^ Robbrecht et al.’s qualitative exploration of medicine registrars and faculty found that registrars recognised the value of LLL, but they desired more ‘structure and guidance’.^6^

To address this gap, this scoping review aimed to explore: How do registrar programmes explicitly teach LLL? Given the heterogeneity in design and scope of available evidence, which precluded meaningful synthesis through a systematic review, a scoping review was chosen to provide a broad overview and inform future curriculum development.

## Methods

The scoping review was conducted in accordance with PRISMA-ScR guidelines (checklist available as Online Appendix 1),^19^ the recommendations of Mak et al.,^20^ and a protocol registered on the Open Science Framework (https://doi.org/10.17605/OSF.IO/YN8CG). As this review synthesised published literature without involving human participants, it was exempt from institutional ethical review. The scoping review methodology enabled a deliberately broad search to capture the range of evidence while remaining feasible, consistent with Arksey and O’Malley’s framework and subsequent refinements.^21^ Postgraduate medical education was defined as formal residency training following an undergraduate medical degree, in line with the standards of the World Federation for Medical Education and the Accreditation Council for Graduate Medical Education.^22,23^ ‘Resident’ refers to a physician in a structured postgraduate training after medical school, and ‘Registrar’ is the equivalent term used in Commonwealth countries for doctors in specialty training.^23,24^ Although related constructs such as self directed learning (SDL), reflective practice and metacognitive skills are purported to promote LLL, they represent distinct constructs and only when these were explicitly framed as strategies promoting LLL were they included. Many PGME studies use these terms without defining them or linking them to LLL as a professional competency. To examine how LLL is conceptualised and intentionally taught, this review included only studies that explicitly used the term ‘lifelong learning’. This approach distinguished studies explicitly conceptualising LLL from those where related constructs (e.g. SDL) may contribute to, but were not explicitly framed as developing LLL. This approach reduced conceptual conflation and ensured findings reflected deliberate LLL educational design rather than inferred associations. Reflexivity guided this decision, selecting investigations that operationalised LLL over those that merely affirmed its importance.

Eligibility criteria included peer-reviewed empirical studies in English that addressed educational strategies for LLL within PGME. Grey literature was excluded to maintain a focus on peer-reviewed empirical evidence. Narrative or opinion-based literature, such as commentaries, editorials, and position statements, as well as education-based studies that explored LLL without a teaching component or without reference to LLL, were excluded. Exclusion followed a stepwise process: firstly, removing off-topic articles; secondly, exclusion by publication type; thirdly, excluding studies that used LLL as a keyword but did not focus on it; and finally, excluding studies that did not examine an educational strategy or registrars.

An online search was conducted across MEDLINE (via SCOPUS^®^), Web of Science Core Collection™, EMBASE (via ProQuest), and the Cochrane Library, guided by the recommendations from Gusenbauer and the National Academies’ standards for systematic reviews.^25,26^ The first search combined MeSH terms ‘lifelong learning’ AND ‘postgraduate medical education’, and the second extended the search to ‘lifelong learning’ and (‘resident’ OR ‘registrar’). The search included all investigations in the databases’ history to the present (27 September 2025 – First search, and 04 November 2025 – Second search). Filters were applied where possible for English language, human participants, and the medical field. The full search strategy is available as Online Appendix 2. Following calibration, gaps in retrieved references necessitated a second search, undertaken in collaboration with a medical librarian to validate and refine search strategies.

Both searches involved two stages: title and abstract screening followed by full-text review. For the first search, two reviewers independently screened all records, achieving 100% agreement on exclusions and 97% agreement on ‘maybe’ or ‘include’ decisions, with discrepancies resolved through discussion. For the second search, one reviewer screened all records, while the second reviewer verified all ‘maybe’ and ‘include’ decisions. Reference lists of included studies were title-screened to identify additional sources. Reviewer roles were complementary: MO, whose research interest lies in LLL, provided content familiarity, while AR provided an impartial perspective.

Data extraction comprised populating a Microsoft^®^ Excel for Mac (version 16.103.1) spreadsheet. Variables included bibliometric data as well as population, speciality, educational strategy, and reported outcomes. Data extracted from included studies were analysed using an iterative process to identify patterns across educational strategies. Similar concepts were grouped into three overarching domains: goal-setting and accountability, reflection with feedback, and EBM, based on thematic convergence observed during synthesis. Methodological quality of included studies was appraised using the Mixed Methods Appraisal Tool (MMAT), which allows evaluation across qualitative, quantitative, and mixed-methods designs.^27^ Finally, stakeholder consultation was undertaken, as recommended by Levac et al.^28^ Consultation was undertaken through email-based review with the Head of the Health Professions Education Unit at the University of KwaZulu-Natal, who provided written expert feedback. The contributions focused on strengthening the justification for the search strategy, which was restricted to explicit ‘lifelong learning’ terminology and helped contextualise results within the PGME context, which are incorporated into the methodology and synthesis of the three themes.

## Review findings

**FIGURE 1:**
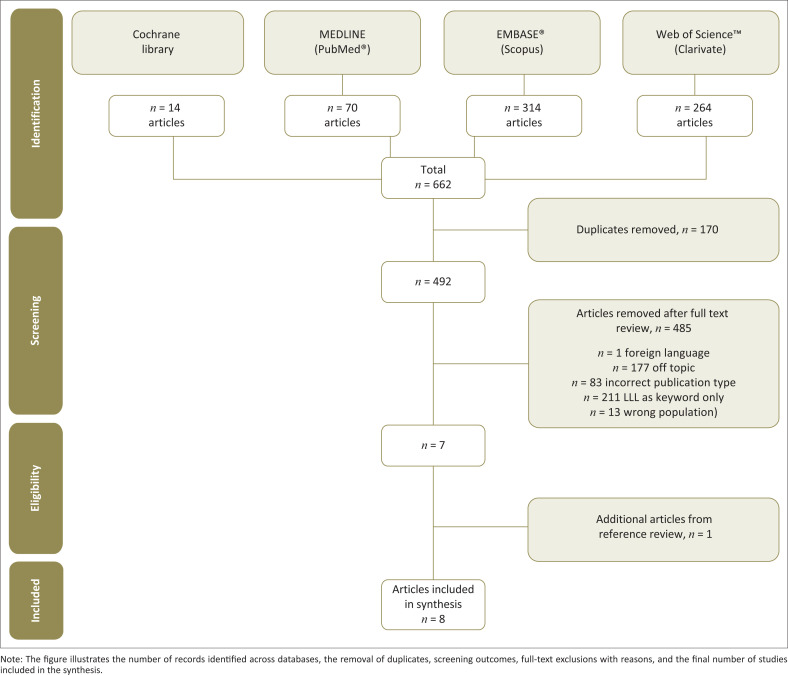
PRISMA-ScR flow diagram of the database search and study selection process.

Eight studies of a total of 492 met the inclusion criteria for this scoping review.^29,30,31,32,33,34,35,36^ Seven were identified through database searches,^29,30,31,32,33,34,35^ and one study was retrieved through a reference list review.^36^ Figure 1 provides a detailed outline of the search and article selection strategy. Most studies were conducted in the United States (*n* = 7), and one in Canada (Table 1). Articles were published between 2009 and 2017. The included research spanned three specialties: paediatrics (*n* = 5), internal medicine (*n* = 2), and psychiatry (*n* = 1). Study designs varied: five were quantitative descriptive surveys, two were mixed-methods studies, and one was a qualitative investigation that included interviews and focus groups. Sample sizes ranged from 23 participants in the qualitative investigation to 992 registrars in a national survey.

**TABLE 1:** Characteristics of included studies examining strategies to foster lifelong learning in postgraduate medical education.

Number	Title	Authors	Year	Journal	Country	Population	Specialty	Study design	Education strategy delivery mechanism	Mixed methods appraisal tool study quality analysis
1	Electronic portfolio use in pediatric residency and perceived efficacy as a tool for teaching lifelong learning^29^	Frank, A. Gifford, K.	2017	BMC (BioMed Central) Medical Education	US	82 paediatric residency programme directors	Paediatrics	Cross-sectional survey	Electronic portfolios	Moderate
2	Pediatric resident and faculty attitudes toward self-assessment and self-directed learning: a cross-sectional study^30^	Li, S.T. Favreau, M.A. West, D.C.	2009	BMC (BioMed Central) Medical Education	US	36 residents, 43 faculty	Paediatrics	Cross-sectional survey	Individualised Learning Plans	Good
3	Factors associated with successful self-directed learning using individualized learning plans during Pediatric residency^31^	Li, S.T. Tancredi, D.J. Co, J.P.T. West, D.C.	2010	Academic Paediatrics	US	992 residents 46 programme directors	Paediatrics and Paediatric Subspecialties	Cross-sectional survey	Individualised Learning Plans	Good
4	Successful self-directed lifelong learning in medicine: A conceptual model derived from qualitative analysis of a national survey of pediatric residents^32^	Li, S.T. Paterniti, D.A. Co, J.P.T. West, D.C.	2010	Academic Medicine	US	992 residents	Paediatrics and Paediatric Subspecialties	Cross-sectional survey	Individualised Learning Plans	Good
5	Assessing residents’ written learning goals and goal writing skill: Validity evidence for the Learning Goal Scoring Rubric^33^	Lockspeiser, T.M. Schmitter, P.A. Lane, L Hanson, J.L. Rosenberg, A.A. Park, Y.S.	2013	Academic Medicine	US	60 residents	Paediatrics	Mixed methods (validation of scoring rubric)	Goal-setting requirement	Good
6	A pilot study exploring the relationship between lifelong learning and factors associated with evidence-based medicine^34^	Mi, M. Halalau, A.	2016	International Journal of Medical Education	US	29 residents	Internal Medicine	Cross-sectional survey	Evidence-based medicine training	Fair
7	Incorporating lifelong learning from residency to practice: A qualitative study exploring psychiatry learners’ needs and motivations^35^	Sockalingam, S. Soklaridis, S. Yufe, S. Rawkins, S. Harris, I. Tekian, A. Silver, I. Wiljer, D.	2017	Journal of Continuing Education in the Health Professions	Canada	23 residents 6 educators 5 early-career psychiatrists	Psychiatry	Qualitative study	Workplace-based learning, Mentorship, Research	Good
8	An Education for Life requirement to promote lifelong learning in an Internal Medicine residency program^36^	Panda, M. Desbiens, N.A.	2010	Journal of Graduate Medical Education	US	27 internal medicine residents	Internal Medicine	Mixed methods (survey and narrative analysis)	Education for Life requirement	Good

Note: Please see the full reference list of the article, O’Connor M, Rocher AGL, Wolf JM, Singaram V, Marais LC. All talk, little action: A scoping review mapping lifelong learning education strategies in postgraduate medical education. J Coll Med S Afr. 2026;4(1), a427. https://doi.org/10.4102/jcmsa.v4i1.427, for more information.

US, United States.

The figure illustrates the number of records identified across databases, the removal of duplicates, screening outcomes, full-text exclusions with reasons, and the final number of studies included in the synthesis.

During the stepwise screening process, the authors observed that the term ‘lifelong learning’ appeared frequently (*n* = 211) as a keyword or competence in PGME literature, highlighting its prominence in the discourse, despite only eight studies meeting our inclusion criteria for detailed analysis. Quality appraisal using the MMAT indicated that the overall methodological quality was moderate to high.^27^ The included studies generally met the criteria for sampling strategy, appropriate measurement, and analysis; however, one study had unclear participant representativeness, and two studies showed a moderate risk of non-response bias.

Across the included studies, educational strategies designed to foster LLL in PGME could be clustered into three main groups. Although the degree of emphasis varied, all eight articles addressed three concepts, either explicitly or implicitly, with considerable thematic overlap across studies. The first group emphasised goal-setting and accountability, using structured plans and tracking systems to help registrars identify learning priorities and monitor progress. The second group focused on reflection, self-assessment, and feedback, aiming to develop intentional habits of critiquing oneself, accepting critique from others, and adapting learning accordingly. The third group focused on EBM and critical appraisal skills, equipping registrars to seek, evaluate, and apply evidence in clinical practice as a foundation for LLL.

### Goal-setting strategies and accountability for lifelong learning

Across the six studies that reported goal-setting strategies to operationalise LLL, authors described the use of structured cycles of goal-setting, monitoring, and revision as ways to incorporate LLL into registrar training. Studies referred to various delivery mechanisms, namely individualised learning plans (ILPs), electronic portfolios, and ISMART-based frameworks (Important, Specific, Measurable, Accountable, Realistic, Timeline).^29,30,31,32,33^ The progress of registrars and the outcomes of these engagements were monitored using tracking systems that could take various forms, including faculty mentorship, rubric scoring, and scheduled reviews.^29,33,35^ Programmes often incorporated workshops to teach goal-writing skills and integrate goals into clinical workflows.^31,33^ Only one programme commented on the efficacy of its strategies to foster LLL.^29^ Frank et al. reported that only 20% of programme directors felt that the e-portfolios were effective in teaching LLL.^29^ Common barriers to goal-setting included time constraints in already dense residency programmes and registrars occasionally struggling with generating realistic goals, which some authors linked to inaccurate self-assessment.^29,30,31,33^

### Reflection, self-assessment, and feedback for lifelong learning

Seven studies described organised opportunities for reflection, paired with external evaluation, to improve the accuracy of self-assessment and learning adaptation.^30,33,36^ Implementation strategies include ILPs, electronic portfolios for documenting reflections and progress, and workplace-based reflection tied to patient care.^29,30,31,32,33^ Faculty formal and informal mentorship reviews enhanced accountability and provided formative feedback.^29,31,32,35^ When supported, reflection and feedback enhanced accountability for learning, while workplace-specific reflection increased motivation and the relevance for practice.^30,31,32^ However, only one of the studies reporting on reflective or feedback-based strategies, the ‘Education for Life’ initiative, reported on the positive impact on residents’ LLL behaviours.^36^ The authors reported that 58% of registrars post-graduation ‘found the “Education for Life” requirement useful for their future continued medical learning’.^36^ Time constraints were a recurring barrier to participation in the programmes, often reducing engagement in these activities when not integrated into clinical workflows.^29,32^

### Evidence-based medicine, critical appraisal, and research to foster lifelong learning

Evidence-based medicine and critical appraisal training were repeatedly linked to LLL and proposed as a means to develop the habits of inquiry and evidence use in three articles.^34,35,36^ Registrars with prior research methods training demonstrated a stronger LLL orientation, better perceptions of EBM environments, and greater self-efficacy.^34^ Implementation strategies include embedding EBM tasks in clinical care, such as point-of-care literature searches, teaching information management, and integrating basic research methods and statistics into curricula.^34,35,36^ Online course modules were recommended to address barriers to access.^35^ Evidence-based medicine-focused learning tied to real patient questions increased the relevance for and motivation of registrars, and supportive environments encourage sustained engagement.^34,35^ Training in critical appraisal alone did not guarantee increased use of literature, however.^34,35^

## Implications and recommendations

This review aimed to identify and map the educational strategies employed to foster LLL among postgraduate medical trainees. While the initial intent was to conduct a systematic review to identify the most effective approaches, the limited volume and heterogeneity of the available literature necessitated adopting a scoping review methodology. This finding highlights that although LLL is widely acknowledged as a fundamental component of professional competence, its practical implementation within PGME remains limited. Numerous studies reference LLL in their titles or keywords, yet few provide structured strategies for its development. The authors contend that this gap introduces the need for intentional educational design and integration to purposefully cultivate LLL, rather than presuming it will occur organically. Lifelong learning extends across the professional lifespan; however, this review focuses on registrar training as one segment of this continuum. While this creates tension between a time-bound training period and an enduring lifelong construct, this stage represents a critical opportunity to develop foundational LLL behaviours.

Among the specialities that have examined educational strategies for fostering LLL, there was a notable absence within surgical disciplines. A previous investigation suggests that an individual’s work environment can encourage the intention to engage in or the engagement in LLL.^37^ Medical education literature often frames surgical training as primarily focused on technical skill acquisition,^13^ with trainees demonstrating a preference for visual and kinaesthetic learning styles.^38^ These characteristics may warrant the adoption of LLL strategies that incorporate multimedia and hands-on learning styles to enhance relevance and uptake in the surgical disciplines.^38^ Potentially, educational approaches for LLL in procedural learning environments should be intentionally tailored, rather than mirroring those in the more cognitively based and less skill-intensive disciplines of psychiatry and medicine.^38,39^

Not only the clinical environment but also the geographic environment may have implications for LLL. Walters emphasises the distinctive social and cultural dimensions of LLL in South Africa, noting that the legacy of educational inequities from the Apartheid era has positioned LLL as a national priority.^40^ This context is of relevance to the authors, given the South African setting of this investigation and its implications for PGME. However, this inequity is not unique to South Africa. Low- and middle-income countries often face substantial clinical workloads and limited supervisory capacity, heightening the need for SDL strategies.^41^ The predominance of studies originating from North America, therefore, raises concerns regarding the global applicability of these findings. Before implementing educational strategies, educators should carefully consider the resource constraints of the context in which they will be applied.

In resource-limited settings, portfolios or ILPs, together with critical appraisal skills (the primary vehicles for developing LLL engagement identified in this review), represent feasible options for fostering LLL. Portfolios can incorporate goal-setting, learning plans, documentation of achievements, and reflective components, which are relatively low-cost but may require institutional support and faculty development. For critical appraisal, previous studies have suggested leveraging free online platforms to promote evidence-based practice in resource-constrained environments.^35^ Nevertheless, all strategies will require careful adaptation to local contexts.

Following a review of current strategies to educate LLL, two persistent challenges remain: firstly, the lack of longitudinal evidence on effectiveness, as few studies have assessed sustained improvement over time; and secondly, the difficulty of integrating these approaches into already burdened training curricula. An emerging development in the literature that may help to address integration challenges is the concept of micro-credentialing, which provides extrinsic incentives to complement the intrinsic motivation underpinning LLL.^42^ Another promising innovation is ‘precision education’, a system designed to efficiently tailor learning to individual needs, fostering agency and continuous improvement in an equitable manner.^43^ The implementation of precision education may not be far off. Desai et al. emphasise the importance of leveraging AI-driven adaptive learning tools to achieve this,^43^ an irony acknowledged by the present authors, given the enduring need for humans to engage in LLL, even in an AI-rich era where such systems still fall short in tasks requiring adaptive reasoning and reflective growth, as discussed in the introduction.

This scoping review has several limitations. Firstly, even with a comprehensive search strategy, some studies may have been missed because many publications address the underlying principles of LLL in education strategies, while using alternative descriptors, rather than the term itself. This variability in terminology points to a significant gap in the field and highlights the need for an accurate, standardised definition of LLL to guide future research and facilitate consistent evidence synthesis. Secondly, the review was restricted to English-language publications, potentially excluding insights from non-English-speaking contexts. Thirdly, as is typical of scoping reviews, the findings are descriptive; therefore, we cannot conclude the effectiveness of the strategies identified. Nonetheless, these findings provide a valuable foundation for developing future educational approaches. Finally, the search yielded no contemporary studies. While concepts such as microcredentialing and precision education are emerging, the absence of recent empirical research suggests that this domain is underexplored rather than rapidly evolving.

## Conclusion

This scoping review identified eight studies that describe strategies to foster LLL during residency. Despite the prominence of LLL in didactic curricula, actual strategies to inculcate it are limited, concentrated in North America, and predominantly found in non-surgical disciplines. The available evidence is limited and does not permit definitive recommendations for practice, highlighting the need for deliberate incorporation and investigation of LLL in postgraduate curricula, to evaluate impact across diverse contexts.
